# Primary Health Care and Family Medicine at the Core of Health Care: Challenges and Priorities in How to Further Strengthen Their Potential

**DOI:** 10.3389/fmed.2014.00037

**Published:** 2014-10-16

**Authors:** Chris van Weel

**Affiliations:** ^1^Radboud University Nijmegen, Nijmegen, Netherlands; ^2^Australian National University, Canberra, ACT, Australia

**Keywords:** primary care, family medicine, health care, challenges, community setting

## Summary

This paper analyses the paradigm shift from the disease to the person with the disease against the background of the changes of health systems toward primary health care. The structural changes from hospital to the community and the specialist to the generalist approach are essential to enable this different approach. As a consequence, any assessment of health status, risks, and needs starts with an engagement with an individual. This engagement is the basis from which diagnostic, preventive, and therapeutic interventions are planned, over time, in the continuous working relation primary health care entertains with individuals and populations. Key to the functioning of primary health care is an ongoing renewal or actualization of this working relation, in this paper referred to as the “initial estimate,” that makes it possible to direct resources to those in highest need and at the same time makes it possible to exempt from costly and risky interventions those who have little to gain from it. This “initial estimate” is a major determinant of cost-effective and efficient healthcare, but there is hardly any insight into the process of how professionals in primary health care come to estimate individuals’ risk. This in turn presents a number of challenges and priorities for primary health care research:

- to build the interaction between practice and researchers in designing and developing tests; secure primary health care research capacity to study and assess tests in the primary health care setting; and secure the implementation of validated tests in routine patient care.
- to open to research the full setting in which patient and professional interact and integrate in this the contribution of diagnostic tests;
- to study professionals’ decision making, including the role of “experience” and “intuition,” mechanisms through which the premonition of something being wrong is coming about and to develop methods to train professionals to apply this in an appropriate way;
- to use these insights to study as well the effectiveness of preventive and therapeutic interventions;
- as the setting in which professionals operate influences the outcome of their performance, make sure that “every” community is connected to the primary health care research capacity.

## Introduction

Many countries in the world live through a transition of their health care systems toward primary health care. In this, the care of patients is to be led from the community rather than the hospital, by a generalist rather than a specialist. This is in itself a fundamental change – a change in the structure of the health care system, in the conditions and facilities under which it operates and professionals and patients engage.

This asks for new structures and models and their implementation is complex in its own right. The development of the concept of “the patient-centered medical home” in the US (1) is a good example of creating approaches that were impossible under the prevailing system. But there is a realistic danger that the transition toward a primary health care structure is seen as just this: replacing the hospital by the community, or the specialist by the generalist, with magical implications attached to this structural solution. Belief in “structural” change, with the generalist-family physician as a super human is often an implicit ingredient of healthcare reform. In this, primary health care continues to operate in the traditions of medicine, with diagnosis and disease as its determining currencies. The orientation on the person is an appreciated and valued add-on with the patient defined to no longer a single disease, but a set of co-existing health problems. But it is highly unlikely that generalists are able to deliver what specialists cannot, only because they practice in another location or there is “*family physician*” written on their front door.

What is essential in the transition of health systems is to secure a different approach, from diseases to individuals with the diseases and populations at risk of the diseases. To be able to achieve this, health care has to be embedded in the environment in which people live and work, run the risk of illness and disease. The health system structural changes are essential exactly because they make a different approach possible, a paradigm shift from the disease to the person with the disease.

And this paradigm shift is the pivotal point in reforming health systems. The challenges of primary health care are to *capitalize* on the working relation it has over time with individuals, families, and communities, in identifying, preventing, and managing health problems. This is the overarching context of continuity and integration of care to provide effective, efficient, safe, and timely health care. However, in the many pressing primary health care research needs, it is an undervalued aspect.

This paper explores the challenges of research to support the successful completion of health care reform as a “structure and paradigm shift.” As will be made clear, the experience of seasoned practitioners in the field may well hold the key and research has to be directed to the systematic exploration of this experience, to create the knowledge that is needed to guide the success of how primary health care, how family physicians, can lead the system.

## Engaging with Individual Patients

Central, in this experience is the encounter between an individual and a health care professional. The encounter takes usually place in the community setting, close to where the individual works and lives. As a consequence, the “community” setting works through in the encounter, which makes primary health care person centered (2) from a people-centered health care approach (3). Phrased in another way, primary health care is directed at the individual with the disease, and with its consequences: for the individual’s ability to function and perform, for the family, for work, and environment. This is the “bio-psycho-social” integration (4) that primary health care is directed at, and this presents the complexity of the professional performance in primary health care. This is an ongoing process of engagement over time, in the continuous working relation primary health care entertains with individuals and populations (5). Key to the functioning of primary health care is the ongoing renewal or actualization of this working relation, in this paper referred to as the “initial estimate” from which further actions evolve.

At the same time, by addressing individuals’ health problems in this integrated approach, primary health care is a key determinant of effective, efficient, costs-containing and affordable, safe, and human health care (6). Strategies to further strengthening of primary health care have to be based on this inherent complexity, and acknowledge that this will lead to diversity in professional performance: the same health problem may have different implications for different individuals and/or different circumstances over time. A better understanding of professional performance in this complexity is essential to assess, test, and implement new interventions. This is where research and science have to make their contribution. This paper analyses this and presents a number of challenges and priorities for primary health care research.

### Professionalism of coping with clinical uncertainty

Primary health care deals with a large variety of important health problems in the community, in all organ systems, in all stages, in all patient groups (7). As it is usually the first point of contact, an important function of primary health care is coming to an understanding of the health problem, of the nature of the disease (“diagnosis”) and of the impact on the person (“illness”). In its early stages, diseases may still have minimal signs and symptoms, which bring an inherent uncertainty to the diagnostic process. A timely diagnosis in this context is often the ability to identify disease with limited means in an early stage.

Family physicians experience growing support in this, from the availability of techniques of near-patient testing: a rapidly expanding set of user-friendly tests to rule-in or rule-out the presence of a specific disease (8). Essential in this is that these techniques have been appropriately tested for their predictive value and reliability (9) before their application in routine primary health care. Mandatory for this is that the test is studied in the actual primary health care setting in which the test will later be used: does the test identify patients with the disease in the early stage when they contact primary health care; and does it distinguish reliably between those with the disease and those with comparable signs and symptoms but due to other causes? These are very specific primary health care needs, in which the negative predictive value of the test, the possibility of ruling-out important disease, and consequently sparing patients’ spurious interventions, is as important as the ruling-in of important disease through the tests’ positive predictive value (10). Diagnostic acuity is directly related to the navigation function of primary health care (11): directing diagnostic, therapeutic, and supportive interventions to those who stand to benefit from it, while excluding those who stand little to gain from it. This is one of the keys to cost-effectiveness of health care, and the resilience of health care systems under the stress of expanding financial pressure (6, 12). This makes the development, analysis, and implementation of diagnostic techniques a formidable research priority for primary health care. From this follows the challenge to build and strengthen the interaction between practice and researchers in designing and developing tests: the primary health care research capacity to study and assess tests asks for the involvement of professionals to define how new tests can be implemented in routine patient care. “Diagnostic research” will definitely find its way into the columns of Family Medicine and Primary Health Care, a section of Frontiers in Medicine.

### Toward the person with the disease

But in strengthening primary health care, it is important to acknowledge that a diagnostic test is part of a more comprehensive primary health care procedure that is directed at the understanding of the person with the disease. Consequently, this means that their analysis should not be valued as a stand-alone intervention but in their contribution to the outcome of the procedure. This is the more important, as “tests” are directed at pathophysiological markers of disease, while symptoms shape the early phase of disease development in which primary health care is involved. As useful example in this context may serve myocardial infarction: in its initial stage (13), ECG or myocardial enzymes may still be normal in the presence of chest pain, feeling unwell, sweating, and other signs and symptoms. Early diagnosis, particularly important to enable early reperfusion intervention, relies under those circumstances on the process of professional interpretation of presented symptoms, rather than on the technology of testing. Diagnostic acuity depends on the interpretation of signs and symptoms to which tests may or may not further contribute. Therefore, “diagnostic research” should consider the comprehensive diagnostic procedure, and position the contribution of test characteristics in this procedure, to strengthen primary health care.

**Figure 1:**
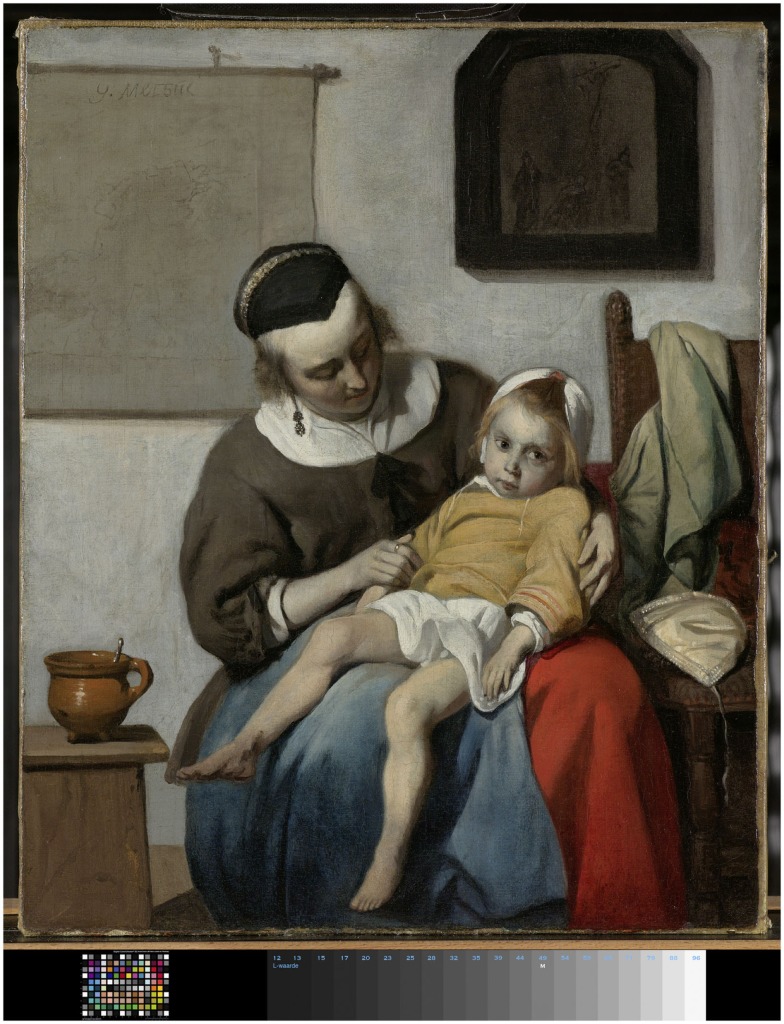
**The sick child of Gabriël Metsu**.

This starts with the encounter with the person with the disease, from which diagnostic cues are derived. To appreciate this, it may be helpful to move to an example from art, in particular, the work of the Dutch seventeenth century painter Gabriël Metsu, the sick child (Rijksmuseum, Amsterdam) (Figure 1). In a simple painting, Metsu presents a child on his mother’s lap, with a bowl of porridge on a small table aside, and virtually all viewers of the work will intuitively agree with its title: this is a sick child. Interesting in this in itself, straight-forward interpretation is how the viewer has come to such a conclusion. What can be observed are the paleness of the child, its listlessness and immobility, its glancing away, not focused on activities in its surroundings. The bowl of food set aside suggests a lack of appetite. This deconstruction of the picture is set against an inner-mind picture of a healthy child, blushing, active, and interacting with its direct environment, from which the notion of “sickness” is constructed. And what the viewer sees is the overarching picture, “sickness,” and not its contributing parts and pieces.

Like Metsu’s viewers, family physicians and other professionals in primary health care deduce diagnostic information in their encounters with their patients, deconstruct, and reconstruct it through an inner-mind comparison. As they often operate in a continuous working relation with a community, they are able to operate three inner-mind comparators: a prototypic healthy person; the picture of the same patient on previous encounters; and a notion of frequent diseases, that is: common in their community. Included in this are the knowledge of the patient’s medical, psychosocial and family history and previous experiences in presenting and coping with illness and disease. In this way, initial diagnostic cues are generated, and the diagnostic procedure is built from it with more detailed information seeking on a “need to” support professional performance. Tests and diagnostic technology, in other words, get their meaning and relevance in this procedure. And this, in turn, presents the actual setting in which diagnostic research gets its relevance. From this follows the challenge to open to research the full setting in which patient and professional interact and integrate in this the contribution of diagnostic tests.

The interaction between patients and professionals is determined by the actual community in which the interaction occurs, the social determinants of health (14). When there is a universal need of primary health care, “a family physician in every community” (15), there is also the need to connect “every” community to the primary health care research capacity. There are limits to which research findings can be translated from one community to another (16) and external validity is an essential marker of quality of primary health care research.

With it comes the need to come to a better understanding of “external” validity and when it is possible to translate research findings to other societal settings. Developing criteria to describe the research setting could be an important first step, to generate empirical data to analyze the translation issue.

This is in all probability the most fundamental challenge as it has to address established practice of an unwarranted implementation of “Western” research findings on non-Western populations. At the same time, it has to face the need to secure conditions of research quality and ethics under which community based research can be embarked on, confidently.

But in the course of this argument, an important transition has been made, that is essential to fully understand primary health care. It can be helpful, again, to return to Metsu and his sick child and the viewer’s construction of an inner-mind picture. The child in Metsu’s painting shows signs, diagnostic cues, of severe distress. This triggers the urgency to identify its source, not as an academic exercise, but for the practical “hands-on” need to intervene. What is noteworthy is that the actual assessment at this point in the diagnostic procedure is not so much about diagnosis or disease, but more about prognosis (9) and the concrete needs and risks of the individual with the disease. Prognostication and risk assessment drive the further diagnostic and therapeutic proceed, and that makes this assessment a core performance of primary health care. It has as a direct consequence that preventive, diagnostic, and therapeutic facilities, including the use of formal tests, are directed at those standing to gain from it, for example, where major disease is suspected. And at the same time, it spares those who do not, the exposure to spurious, costly testing. This approach is one of the determinants of the effectiveness, efficiency, and costs of health care. And from it follows a challenge of primary health care research: not only diagnostic performance but also preventive and therapeutic proceeding, in fact, the integral performance, is embedded in and follows from the setting in which patient and professional interact and integrate. It is this interaction that builds and maintains the trust in which patients allow their care to be provided. Through re-engagement over time, this relation of trust can be reconfirmed and strengthened, but has to be deserved, with a key role of how the professional comes to an “initial estimate.” This has therefore to be the research setting as well to study the outcome of primary health care.

Prognostication and risk assessment are a great good for health care, but at the same time there is a big problem. To a large extent, the generation of diagnostic cues is a subconscious process, which has hardly been formally researched. It is often linked to “intuition,” or “experience” and in Dutch family medicine jargon this has been coined as what can be best translated in English “a premonition of something being wrong” (*pluis/niet-pluis gevoel*). This may aptly summarize the inner-mind process but lends mythical dimensions to it that do little to come to an understanding of the process behind it. Therefore, the “premonition of something being wrong” presents itself as a high priority domain to gain a better understanding of professional performance in the complexity of primary health care. While its appropriate application may lead to substantial gains in health, unsuitable practice may lead to missed opportunities of prevention or diagnosis, delays in treatment, and medicalization. A major challenge of primary health care research is to get into the mechanisms of how the premonition of something being wrong is coming about and to develop methods to train professionals to apply this in an appropriate way.

This reconnaissance has identified a number of challenges of research in the primary health care setting. These challenges are closely related to the values of primary health care (17, 18): community based (3), person-centered (2), integrating the bio-psycho-social domains (4), continuity of care (5), and generalism (18) with a seamless integration of empowerment, prevention, health promotion, diagnosis, treatment, and support. This offers a research approach to analyze the promotion of health and the way health and welfare are connected.

Central in facing these challenges is the connection of research to primary health care practice: building a research capacity of professionals from the field as researchers and networking between research institutes and practices – practice-based primary health care networks (19). This stresses the need to connect in the research endeavor the domains of health problems and of health care systems: the implementation of primary health care policy (20). Together with a better understanding of the diagnostic, preventive, and therapeutic proceeding of patients with primary health care health problems comes the importance of building the primary health care structure in the health care system. In the end, the ultimate challenge is to secure health care systems in every region of the world that are built on primary health care and fully benefit from it for its populations (20, 21).

In conclusion, key challenges and priorities for primary health care research are:

- to build the interaction between practice and researchers in designing and developing tests; secure primary health care research capacity to study and assess tests in the primary health care setting; and secure the implementation of validated tests in routine patient care.
- to open to research the full setting in which patient and professional interact and integrate in this the contribution of diagnostic tests;
- to study professionals’ decision making, including the role of “experience” and “intuition,” mechanisms through which the premonition of something being wrong is coming about and to develop methods to train professionals to apply this in an appropriate way;
- to use these insights to study as well the effectiveness of preventive and therapeutic interventions;
- as the setting in which professionals operate influences the outcome of their performance, make sure that “every” community is connected to the primary health care research capacity.

## Conflict of Interest Statement

The author declares that the research was conducted in the absence of any commercial or financial relationships that could be construed as a potential conflict of interest.
